# New Method of Devitalizing the Pulp

**Published:** 1903-05

**Authors:** 


New Method of devitalizing the Pulp.—In the December, 1902, number of *L'Odontologia* appears a paper by Jaime D. Losada, on "A New Method for devitalizing the Pulp."

The writer, speaking in the plural, claims priority in the method of pulp devitalization by pressure, referring to an article published in *L'Odontologia* for July, 1899, and a demonstration later in the same year before the Dental Congress in Barcelona.

In refuting the theory that arsenic devitalizes by strangulation, the writer refers to the work of Arkövy and Miller, and states that

---

tissue is destroyed by arsenic where strangulation is impossible, and that if this theory were true, filaments of partially extirpated pulps would not remain vital when a portion has been removed after arsenic.

The action of arsenic is progressive, beginning at the point of application and extending gradually. When the pulp is not exposed it penetrates by the dental tubuli and odontoblastic cells. Arsenic acts as a paralyzant, causing hyperæmia, affecting the vasomotor nerves and destroying nutrition.

To relieve pulpitis and prevent its recurrence, immediate extirpation with an anæsthetic and pressure is satisfactory when possible, but the immediate extirpation of the pulp in tortuous canals of multi-rooted teeth is not practicable, and in such cases "arsenical devitalization by pressure" is offered.

Anæsthesia is first obtained with cocaine by pressure, then arsenical fibre, or trioxide of arsenic in solution or powder, is applied with cotton saturated with oil of cloves, followed with raw vulcanite and burnisher, repeating five or six times in ten minutes, thus obtaining the therapeutic action in a short time. The arsenic may be left for a few hours a day to several days. The quantity of arsenic necessary to devitalize a pulp is so infinitely small as not to have been determined, notwithstanding the careful work of Dr. James Truman, of Philadelphia, in this regard.

In twenty cases two were unsatisfactory, one of pulp nodules and an upper molar where the buccal canals were almost obliterated with contents sensitive, while in the palatal root the pulp was insensible.

P. B. McC.

